# Microbiology 2.0–A “behind the scenes” consideration for artificial intelligence applications for interpretive culture plate reading in routine diagnostic laboratories

**DOI:** 10.3389/fmicb.2022.976068

**Published:** 2022-08-04

**Authors:** B. DeYoung, M. Morales, S. Giglio

**Affiliations:** LBT Innovations, Adelaide, SA, Australia

**Keywords:** artificial intelligence, laboratory automation, machine learning, laboratory software, culture plate reading

## Abstract

Laboratory automation with Artificial Intelligence (AI) features have now emerged into routine diagnostic clinical use to interpret growth on agar plates. Applications are currently limited to urine samples and infection control screens, yet some of the details around the development of algorithms remain entrenched with AI development specialists and are not well understood by laboratorians. The generation of algorithms is not a trivial task and is a highly structured process, with several considerations needed to develop the appropriate data for specific intended uses. Understanding these considerations highlights the limitations of any algorithm created and informs better design practices so that algorithm objectives can be thoroughly tested prior to routine use.

Culture plate interpretation requires many years of training (Van Eldere, 2005) and it is only through frequency of plate observations that the most common and rare organisms can be studied in a clinical context, and thus competency achieved through repetition. Despite the years of training and professional desire to be 100% accurate 100% of the time, this is unfortunately not realistic. There is a fundamental risk with any manual interpretation of a culture plate that the interpretation is incorrect, especially when looking for single colonies amongst commensal flora (Gammel et al., 2021). Non-consensus results between microbiologists have also been reported by Glasson et al. (2016) who found that agreement between microbiologists for colony morphology is 97.5% when reading MacConkey agar but only 87.5% when reading blood agar. Similarly, Brenton et al. (2020) identified that microbiologists agree 88.6% of the time when enumerating bi-plate growths. Who would have thought microbiologists can’t agree?

Artificial Intelligence (AI) and some advanced digital imaging applications offer the ability to standardize some, but not all, aspects of interpretive microbiology. AI algorithms evolve from many inputs which are computationally modeled to determine the output with the highest likelihood of a correct result for a given application (Ford and McElvania, 2020). For culture plate interpretation, the considerations for what drives algorithm development is not trivial, but indeed a well-considered and structured process, generally involving work across multiple functional groups that include clinical leads, microbiologists, engineers, AI specialists, and software developers. Producing a reliable algorithm that is thoroughly evaluated and fit-for-purpose is practically difficult and requires stringent conditions and processes for establishing the right dataset for inputs/training, and ultimately how to test/validate/verify algorithm performance when compared to a truth state.

Indeed, there is no play book here, however, the performance of an algorithm is entirely dependent on the quality of the data used and that the source of the training data is critical for determining the final application or intended use. In this respect, it is important to note that the more generalization of the model for classification, the broader its application, and the higher the likelihood of success with respect to performance accuracy over the usual laboratory conditions. The FDA have recently released guidance principles for Good Machine Learning Practice for Medical Device Development (^1^, accessed February 18, 2022) which provides an over-arching roadmap for development of AI-enabled software in devices, clearly applicable to intended applications for culture plate reading. At the core of this document is patient risk; the device which uses AI must not introduce any additional risk for the safety of the patient. Data, data management, and measurable performance of deployed algorithms are critical to ensuring patient risk is minimized.

Defining the test or the intended use of the algorithm is especially important. How will the algorithm be useful, and thus what needs to be considered in design? Sometimes the seemingly innocuous details will have a major impact on algorithm performance unless upfront consideration is given. Using urine cultures as an example, variations that occur across testing laboratories at the pre-interpretation stage include specimen collection and the use of preservatives, inoculation volumes, inoculation methods (metal loops, plastic loops, pipette, magnetic balls), streaking methods (fishbone, quadrant, user-defined), label types and materials (paper, plastic), barcode types, barcode size, barcode printing, and position of labels. Of course, the urine needs to be deposited onto an agar and the algorithm must be developed with a specific manufacturer (or manufacturers) in mind, as subtle differences in media composition occur, even within the same media type across manufacturers. Somewhat ironically, AI is especially sensitive to subtle changes that are usually not discernible by humans, but the algorithm is effectively broken or compromised. The use of controls to ensure system integrity with no component failure is especially critical in AI systems to assure consistent algorithm performance. What this means is that once the algorithm is developed, it is developed for a very defined set of variables that must remain fixed, and the system must be in strict control. Indeed, the method of image acquisition must also remain fixed for any given AI system, as changing the lighting, camera, lighting and camera angles will all result in “new” images that will affect algorithm performance. Additionally, introduction of any new streaking method, for example, will need detailed investigation to examine any change in overall performance, and it may well be the case that algorithm remediation (or re-development) is required, followed by re-validation activities.

Currently, the PhenoMatrix™ (Copan Italia, Brescia, Italy) application provides an assessment of an agar plate in conjunction with expert rules where the user must accept the decision from the algorithm to proceed with reporting or further work, with published studies covering group B *Streptococcus* and *Streptococcus pyogenes* (Van et al., 2019; Baker et al., 2020). The APAS^®^ Independence (Clever Culture Systems) is currently the only system with FDA clearance as a class II medical device which is diagnostic for a negative no growth result from urine samples (Glasson et al., 2017), and for no significant growth when used in MRSA detection (Gammel et al., 2021). In these cases, human intervention is only required for significant growths. Recently, a deep convolutional neural network was developed using images for urine samples captured by BD Kiestra. Retrospective images were assessed offline and compared the Standard of Care (SoC) results to the algorithm classification (Alouani et al., 2022). The authors reported 98% accuracy for the overall model, but also highlighted the differences in models produced from two independent data sets. Having said that, the model was collectively trained with over 100,000 images over a 2-year period which is quite an impressive data set that would ultimately have a large degree of generalization to account for the day-to-day nuances one might expect in laboratory workflows. However, it is unclear how this offline system might be implemented into routine laboratory procedures, and the authors also recognize that maintaining such a system is a “large undertaking.”

The expectations on AI applications are continually shifting, perhaps ambitiously. Given the applications described above, there is a good reason to expect more. From an industry and regulatory perspective, however, only the intended uses supported from the media manufacturer can be supported from AI applications, unless otherwise justified and substantive evidence is presented for additional claims support. This means strict adherence to specimen type and incubation conditions. Considering that the media was initially developed for human assessment using the well-trained microbiologist eye, and not an advanced imaging system with or without AI, this paradigm should shift to allow advancements in technology to challenge the status-quo of decreasing media read-times especially, as decreased read-times assist with laboratory turnaround times and thus patient management. Perhaps with an increasing body of evidence to support any proposed indications for use, either regulatory positions should be challenged, or manufacturers of media and industry AI-development specialists should work together to advance the field with new indications for use.

While the use of AI in the laboratory setting can be highly beneficial there are still some issues to be addressed. The first being phenotypically distinct single organism polymorphisms that may be interpreted by AI as separate organisms, as may also be the case for a human assessment, as well as small colony variant categorization. As detailed earlier, the broader the inputs, the greater the generalization of the model, and the higher the likelihood of algorithm accuracy. In that respect, understanding and planning around these design constraints is critical for ultimate deployment of algorithms. Additionally, expecting an AI system to correctly categorize “contamination” is a difficult task as often this again seemingly innocuous decision is dependant on years of experience and understanding the specimen type and the full clinical picture with detailed clinical histories. In this respect, a fully integrated AI-LIS system where all data is available may assist, but it is currently not possible to gather this granular detail needed to make this assessment reliable.

It is clear that laboratorians’ thirst for knowledge in AI is high, and as the number of detailed publications continue to emerge both education and training in AI strategies is necessary in order to better understand some of the limitations of the technology. This is especially important when considering implementation into routine workflow and ensuring mitigations are in place to effectively remove any false negatives calls from automated systems. It is also somewhat risky to expect algorithms to “learn” as they go and adapt sample by sample in an uncontrolled manner. Certainly, the fixed algorithm approach has generated enough evidence to demonstrate that the use of these technologies is low risk to patients, demonstrated by high sensitivities/positive percent agreements (PPA) and high negative predictive values (Faron et al., 2016; Brenton et al., 2020; Uwamino et al., 2022). It may also be a stretch to think that AI will reliably identify organisms in a reproducible manner, *without* any additional confirmatory work (unless indicated by media manufacturers). Although there is a natural progression to attempt this using chromogenic agars (and certainly categorization of colors is possible), non-differential agars will be a challenge.

“*The sad thing about artificial intelligence is that it lacks artifice and therefore intelligence.”*–Jean Baudrillard. AI applications in this field cannot be made unless the intelligence, skill, and foresight of microbiologists are driving the development in the required direction. The surface has only been scratched, and we are all part of the AI advance in our field.

## Data availability statement

The original contributions presented in this study are included in the article/supplementary material, further inquiries can be directed to the corresponding author.

## Author contributions

All authors listed have made a substantial, direct, and intellectual contribution to the work, and approved it for publication.

## Footnotes

1
https://www.fda.gov/media/153486/download
